# Circular RNA Circ_0008043 promotes the proliferation and metastasis of hepatocellular carcinoma cells by regulating the microRNA (miR)-326/RAB21 axis

**DOI:** 10.1080/21655979.2022.2044260

**Published:** 2022-02-27

**Authors:** Kangjun Zhang, Taishi Fang, Dong Zhao, Fulan Cen, Xu Yan, Xin Jin

**Affiliations:** a Hepatic Surgery Department, The Third People’s Hospital of Shenzhen, Shenzhen, Guangdong Province, China; b Department of Intensive Care Unit, The Third People’s Hospital of Shenzhen, Shenzhen, Guangdong Province, China

**Keywords:** circ_0008043, hepatocellular carcinoma, miR-326, RAB21, proliferation, invasion

## Abstract

Circular RNAs (circRNAs) are non-coding RNAs with covalently closed structures that modulate the progression of hepatocellular carcinoma (HCC). Here, we explored whether circ_0008043 regulated the biological function of HCC cells. Quantitative real-time polymerase chain reaction (qPCR) was used to detect circ_0008043, microRNA (miR)-326, and RAB21 levels. Expression of E-cadherin, N-cadherin, and vimentin was assessed using qPCR. Cell proliferation, migration, and invasion were evaluated using 3-(4,5-Dimethylthiazol-2-yl)-2,5-diphenyltetrazolium bromide, colony formation, and transwell assays. Xenograft tumors were used to evaluate cell growth *in vivo*. The interaction between miR-326 and circ_0008043 or RAB21 was assessed using dual-luciferase reporter analysis and RNA pull-down analysis. The data illustrated that circ_0008043 and RAB21 were highly expressed, while miR-326 was expressed at less levels in HCC tissues and cells. Interfering with circ_0008043 suppressed cellular proliferation, migration, invasion, and cell growth. Circ_0008043 was confirmed to be an miR-326 sponge that targets RAB21. Rescue experiments showed that inhibiting miR-326 abrogated the effect induced by knockdown of circ_0008043, and overexpressed RAB21 abolished the effect induced by miR-326 overexpression. In summary, silencing of circ_0008043 impeded HCC progression by regulating the miR-326/RAB21 axis. These data suggest that circ_0008043 may have clinical value in the treatment of HCC.

## Graphical abstract

**Figure:**
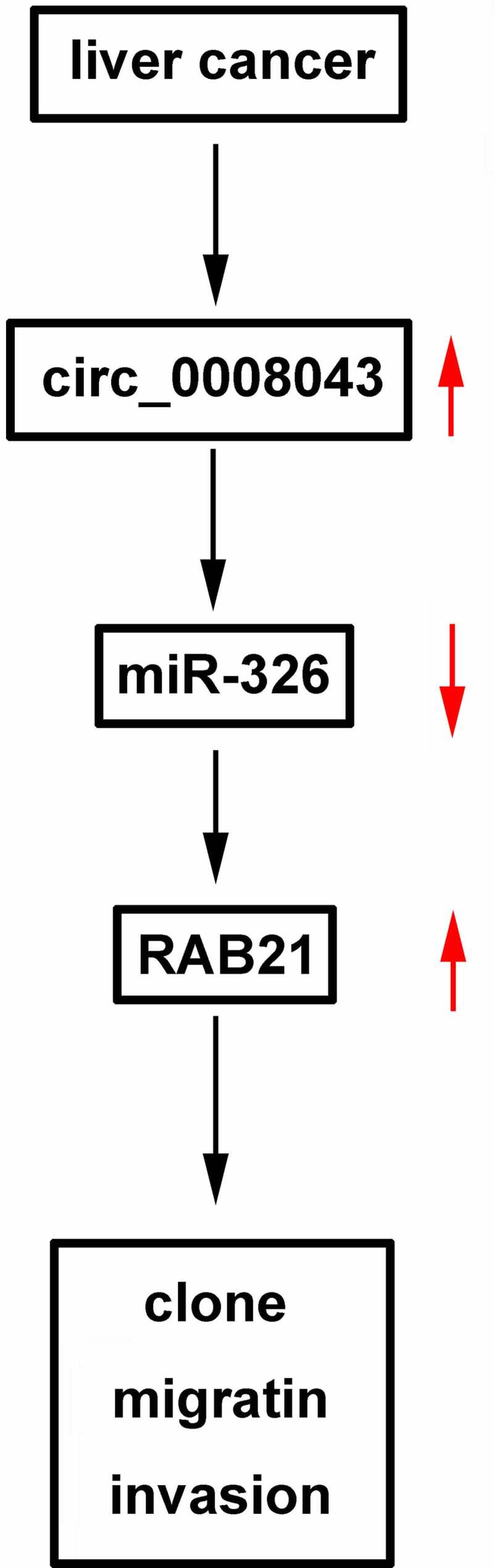

## Introduction

Primary liver cancer is a malignant tumor that commonly originates from hepatocytes and intrahepatic bile duct cells and is the No. 2 leading cause of cancer related death [1]. Hepatocellular carcinoma (HCC) is the main type of highly heterogeneous liver cancer [2]. The incidence of HCC has increased in recent decades [3]. Early stage HCC is usually asymptomatic, while HCC at mid- to late-stage results in a poor long-term survival rate [4]. Current therapies such as surgery, chemotherapy, and transplantation are not effective in treating advanced HCC [5]. Moreover, distant metastasis, drug side effects, and drug resistance limit therapeutic and prognostic effectiveness [6,7]. Thus, investigating more targets may help clarify the pathogenesis of HCC and improve therapeutic efficacy.

Circular RNAs (circRNAs) are newly discovered noncoding RNAs that are widely found in almost all species. They have covalently closed structures formed using a reverse splicing method [8]. CircRNA has gradually become a new star in terms of disease research because of its conservative and stable characteristics. Most of the circRNAs are mainly located in the cytoplasm, and they function as microRNA (miRNA) sponges or act by combining with of RNA-binding proteins [9,10]. Growing evidence suggests that circRNAs are aberrantly expressed in numerous cancers, acting as tumor suppressors or promoters [11,12]. In HCC, dysregulation of circRNAs is involved in tumor occurrence and development, regulating growth, metastasis, and chemoresistance of tumor cells [13]. However, the role of most circRNAs in HCC is still not understood.

CircRNAs are commonly act as competing endogenous RNAs (ceRNAs) to bind miRNAs, and further regulate gene expression [13]. MiR-326 has 20 nucleotides that are coded by intron 1 of chromosome that originally identified as a neural‐specific miRNA [14]. However, recent data reported that miR-326 is significantly associated with the initiation and progression of diseases, such as malignant tumors, inflammation, and cardiovascular diseases [^15–17^]. MiR-326 is usually downregulated in cancers, such as breast cancer, lung cancer, gastric cancer, as well as HCC [^18–21^]. However, the relationship between circ_0008043 and miR-326 in HCC remains unclear.

RABs are critical regulators of cell growth, cytoskeleton assembly, and membrane transport. Rab21 maintains its structure and function in the Golgi apparatus [22]. Interestingly, some members of the RAB family such as RAB21 and RAB25 are closely linked with tumor cell invasion and migration [23]. RAB21 is dysregulated in cancer. Its levels were increased in glioma, pancreatic cancer, but downregulated in prostate cancer [^24–26^]. However, the expression of RAB21 and the function in HCC need explored.

In the present study, differentially expressed circRNAs were identified in HCC cells. Circ_0008043 is one of the differentially expressed circRNAs. We aimed to investigated the biological functions and molecular mechanisms of circ_0008043 were further studied. We hypothesized that silencing circ_0008043 inhibited HCC cell proliferation, migration, invasion, and metastasis. Mechanistically, circ_0008043 regulated cellular processes via the miR-326/RAB21 axis. The goal of this study is to provide theoretical basis for the potential of circ_0008043 in clinical therapy of HCC.

## Materials and methods

### Microarray analysis

Gene microarray GSE155949 for circRNA expression profile was downloaded from the GEO database. The R language package was used to screen differentially expressed circRNAs. The criteria for differentially expressed circRNAs were |log2(FC)| > 2 and P < 0.05.

### Clinical specimens and cell lines

Fresh HCC tissues and paracancerous non-tumor tissues were obtained from patients undergoing HCC surgery. All the tissues were frozen in liquid nitrogen, and then stored at −80°C until use. This study was approved by the Ethics Committee of the Third People’s Hospital of Shenzhen. Written informed consent was obtained from all patients. Clinical information of all patients was listed in Table 1.

**Table 1.** Clinicopathologic characteristics of study subjects

Clinicopathologic characteristics	*n*	Low (n = 62)	High (n = 58)	*p*-value
Age (years)				0.8594
< 50	29	16	13	
≥ 50	34	18	16	
Sex				0.4222
Male	27	13	14	
Female	36	21	15	
AFP				0.0203*
<20 ng/ml	25	9	16	
≥20 ng/ml	38	25	13	
Tumor size				0.0365*
<50 mm	28	11	17	
≥50 mm	35	23	12	
Differentiation				0.0416*
I/II	26	18	8	
III/IV	37	16	21	
Tumor number				0.0593
1	31	13	18	
≥2	32	21	11	
Distant Metastasis				0.0394*
YES	37	27	16	
NO	83	7	13	

Note: AFP, α-fetoprotein.

HCC cell lines (Hep3B, SNU449, Huh7, Focus, MHCC97H, HA22T, and HCCLM3) and normal THLE3 cells were purchased from the Chinese Scientific Academy (Shanghai, China). The cells were maintained in DMEM (Gibco, USA) supplemented with 10% fetal bovine serum (FBS) (Gibco) at 37°C and 5% CO_2_.

### Quantitative real-time polymerase chain reaction (qPCR)

The MiRNeasy Mini kit (Qiagen, Valencia, CA, USA) was used to isolate total miRNA, and an RNeasy mini kit (Qiagen) was used to isolate total RNA. Reverse transcription was performed using the 1^st^ Strand cDNA Synthesis SuperMix for qPCR (Yeasen, Shanghai, China). Subsequently, qPCR was conducted using Hieff® qPCR SYBR Green Master Mix (Yeasen) on a CFX96 qPCR system (Bio-Rad, USA) under the following conditions: 95°C for 5 min, 40 cycles of 95°C for 10s, and 60°C for 30s. The 2^−ΔΔCt^ method was used to detect relative expression (fold changes). GAPDH and U6 served as the internal controls.

### Rnase R and actinomycin D assays

After total RNA isolation, 10 U Rnase R (Lucigen, USA) was incubated with 2.5 μg RNA for 20 min. In addition, Focus and HA22T cells were incubated with actinomycin D (2 μg/mL) for 0, 4, 8, 12, and 24 h. Circular and linear RNA levels were examined using qPCR.

### Cell transfection

Short hairpin RNA (shRNA)-circ_0008043, sh-negative control (nc), mimic and inhibitor of miR-326, nc mimic and inhibitor, RAB21 overexpressing vector, and empty vector (Genepharma, Shanghai, China) were transfected into Focus and HA22T cells using Lipofectamine 3000 (Invitrogen, USA). The cells were cultured at 37°C for 48 h and qPCR was conducted to test the transfection efficiency.

### 3-(4,5-Dimethylthiazol-2-yl)-2,5-diphenyltetrazolium bromide (MTT) assay

Cell viability was performed as previously described [27]. Cells cultured in 96-well plates were incubated for an appropriate time (0, 0.5, 1, 2, and 3 days). Subsequently, the cells were incubated with MTT solution (5 mg/mL; 20 μL) for 4 h. DMSO (200 μL) was added to the solution. absorbance values were detected using a microplate reader (Thermo Fisher Scientific, Waltham, MA, USA).

### Colony formation analysis

Colony formation analysis was performed as previously described [27]. Cells digested using trypsin were plated in 6-well plates at the concentration of 50 cells/well and incubated at 37°C. The medium was replaced every 3 days. Fourteen days later, the colonies were immobilized with 4% formaldehyde, stained with 0.1% crystal violet, and counted under a microscope.

### Migration and invasion assay

Cell migration and invasion were assessed by transwell assay [28]. Transwells pre-coated with Matrigel (BD Biosciences, USA) were used to assess invasion capability, while the inserts without Matrigel were used to analyze migration ability. Complete medium (0.6 mL) was added to the lower chambers. Serum-free medium (0.2 mL) supplemented with cells was added to the top wells. After 24 h, the non-migrated or non-invaded cells were wiped using sterile swabs. The migrated or invaded cells were immobilized with methanol, stained with 0.1% crystal violet, and visualized under a microscope.

### Xenograft tumors

BALB/c nude mice (female, 6–8 weeks old), obtained from Shanghai SLAC Laboratory Animal Co., Ltd. (Shanghai, China), were divided into adenovirus (Ad)-sh-nc and Ad-sh-circ_0008043. All mice were kept under standard conditions with free food and water. Four mice were included in each group. Sh-nc and sh-circ_0008043 fragments were inserted into the adenovirus and transfected into HA22T cells. The transfected cells (1 × 10^6^ cells) were injected into the backs of the mice. Tumor size was detected every 1 week for 4 weeks. The volume was calculated using the following formula: 0.5 × length × width^2^. Mice were sacrificed, and tumors were excised. Tumor weight was measured. The animal study was approved by the Ethics Committee of the Third People’s Hospital of Shenzhen. The criterion of the animal study was in accordance with the Guide for the Care and Use of Laboratory Animals.

### Dual-luciferase reporter analysis

We cloned the possible binding sites of circ_0008043 and RAB21 to miR-326 into the pmir-GLO plasmid (Promega) to construct wild-type (wt)-circ_0008043 and wt-RAB21. Mutant (mut) sequences of circ_0008043 and RAB21 were designed and cloned into the pmir-GLO plasmid to construct mut-circ_0008043 and mut-RAB21. Focus and HA22T cells were co-transfected with wt-circ_0008043/wt-RAB21 or mut-circ_0008043/mut-RAB21 and mimic or NC mimic using Lipofectamine 2000. Luciferase activity was detected using the dual Glo Luciferase Assay System (Promega) after 48 h.

### RNA pull-down assay

The cells were transiently transfected with biotin-labeled miR-326 and nc. The cells were harvested after 48 h. After lysis, the cell lysates were incubated with Dynabeads M-280 Streptavidin (Invitrogen) at 4°C for 3 h. Subsequently, the beads were washed with lysis washing buffer. qPCR was conducted to examine the enrichment of circ_0008043 and RAB21.

### Statistical analysis

The experimental data were acquired from three independent replicates. Data analysis was performed using GraphPad Prism 6 software, and the results are shown as mean ± standard deviation (SD). Student’s *t*-test was used to compare the two groups, and one-way analysis of variance was used to compare multiple groups. Statistical significance was set at P < 0.05.

## Results

In this study, we explored the role of circ_0008043 and potential molecular mechanism. We evaluated cell proliferation, migration, invasion, and epithelial-mesenchymal transition (EMT) of HCC cells, and tumor growth *in vivo*. Knockdown of circ_0008043 inhibited cell biological behaviors by regulating the miR-326/RAB21 axis. The data may provide a new insight for circ_0008043/miR-326/RAB21 axis in HCC treatment.

### Circ_0008043 is highly expressed in liver cancer

**Figure 1.**
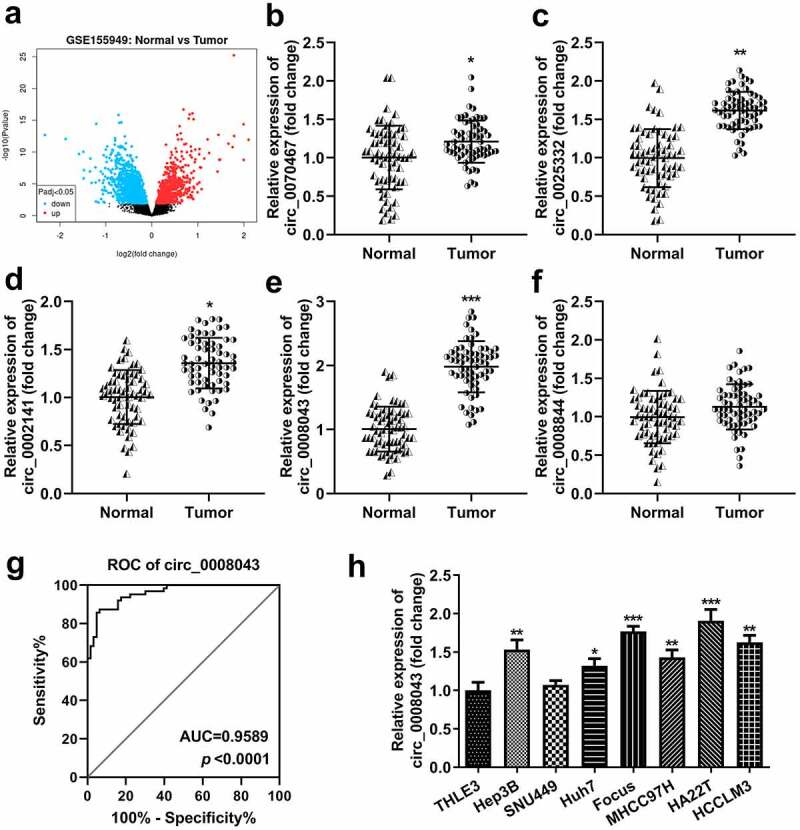
Circ_0008043 is highly expressed in liver cancer. (a) The differentially expressed circRNAs in HCC tissues were evaluated using microarray analysis and visualized by volcano plots. Blue points are downregulated circRNAs, and red points are upregulated circRNAs. qPCR was performed to detect the (b) circ_0070467, (c) circ_0025332, (d) circ_0002141, (e) circ_0008043, and (f) circ_0008844 expression in HCC and adjacent non-tumor tissues. (g) ROC curve for circ_0008043. (h) qPCR was conducted in HCC cells (Hep3B, SNU449, Huh7, Focus, MHCC97H, HA22T, and HCCLM3) and normal THLE3 cells. *P < 0.05. **P < 0.01. ***P < 0.001.

First, we screened differentially expressed circRNAs in the tissues of HCC patients. Volcano plots showed numerous upregulated and downregulated circRNAs (Figure 1(a)). Then, circRNA levels were detected using qPCR. The results indicated that circ_0070467, circ_0025332, circ_0002141, and circ_0008043 levels significantly increased in tumor tissues, whereas circ_0008844 was not abnormally expressed between tumor and normal tissues (Figure 1(b–f)). The receiver operating characteristic (ROC) curve showed that the area under the curve (AUC) was 0.9589 and P < 0.0001 (Figure 1(g)). Additionally, circ_0008043 expression was significantly elevated in HCC cell lines, including Hep3B, Huh7, Focus, MHCC97H, HA22T, and HCCLM3 cells, compared to normal THLE3 cells, while there was no significant difference between THLE3 and SNU449 cells (Figure 1(h)). Moreover, we found abnormal expressed circ_0008043 is closely related to α-fetoprotein (AFP), tumor size, differentiation, and distant metastasis, but not linked to age, sex, and tumor number (Table 1).

### Circ_0008043 is a stable circular transcript

**Figure 2.**
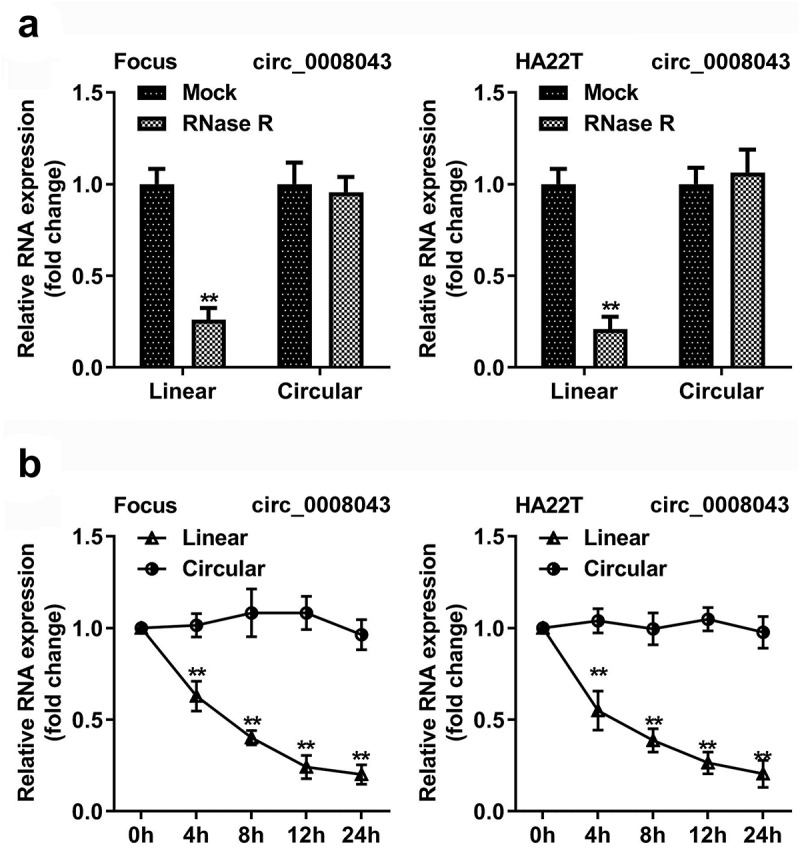
Circ_0008043 is a stable circular transcript. (a) qPCR was used to analyze the resistance of circular and linear RNA to RNase R. (b) The stability of circular and linear RNA was assessed using qPCR after actinomycin D treatment. **P < 0.01.

As illustrated in Figure 2(a), circular RNA was resistant to Rnase R, but the corresponding linear RNA was degraded by Rnase R (Figure 2(a)). In addition, after HCC cells treatment with actinomycin D, the half-life period of circular RNA was more than 24 h, but the half-life period of the linear RNA was between 4 and 8 h (Figure 2(b)).

### *Circ_0008043 knockdown inhibits the proliferation, migration, invasion, and EMT of HCC cells* in vitro

**Figure 3.**
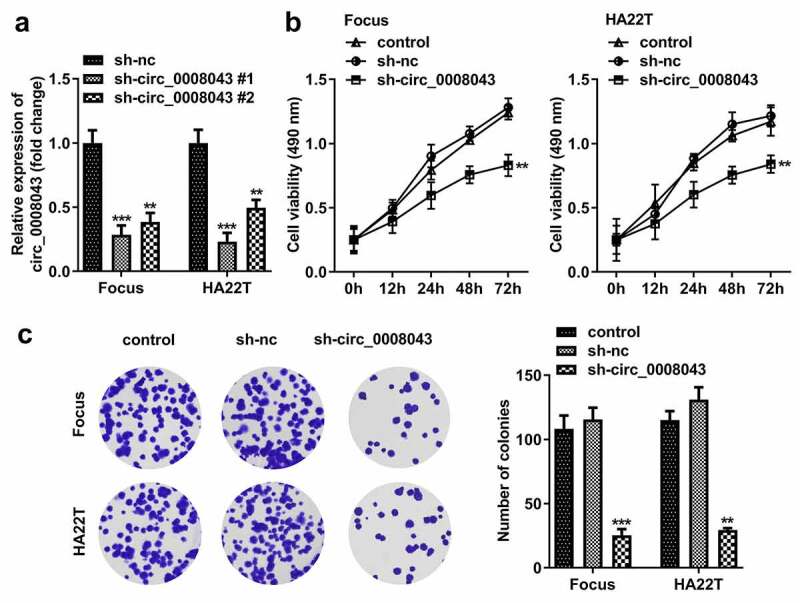
Interfering of circ_0008043 inhibits HCC cell proliferation. (a) The circ_0008043 expression was detected using qPCR post-transfection. Cell proliferation was analyzed by (b) MTT assay and (c) colony formation assay. **P < 0.01. ***P < 0.001.

**Figure 4.**
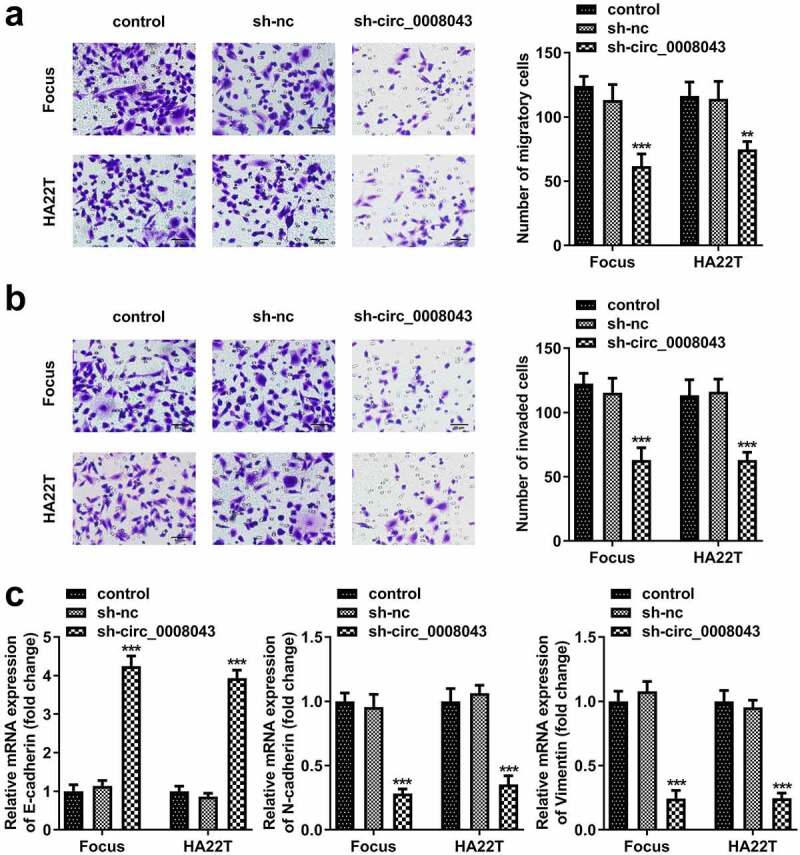
Interfering of circ_0008043 inhibits HCC cell migration and invasion. (a) Transwell assay without Matrigel was performed to evaluate cell migration. (b) Transwell assay with Matrigel was conducted to assess cell invasion. (c) qPCR was utilized to measure E-cadherin, N-cadherin, and vimentin expression. **P < 0.01. ***P < 0.001.

To explore the function of circ_0008043, Focus and HA22T cells were transfected with sh-circ_0008043 #1 and sh-circ_0008043 #2, and the levels of circ_0008043 were significantly reduced after transfection (Figure 3(a)). Knockdown of circ_0008043 markedly suppressed cell viability and colony formation compared to the sh-NC group (Figure 3(b,c)). In addition, knockdown of circ_0008043 markedly suppressed cell migration and invasion (Figure 4(a,b)). Moreover, silencing of circ_0008043 induced the upregulation of E-cadherin and downregulation of N-cadherin and vimentin (Figure 4(c)).

### *Interfering with circ_0008043 impedes tumor growth* in vivo

**Figure 5.**
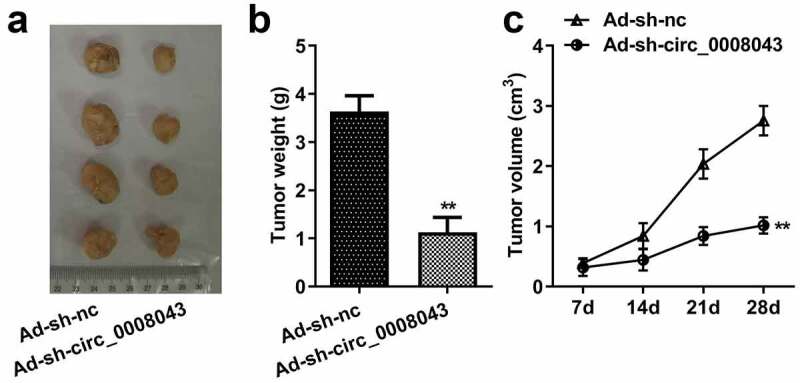
Interfering of circ_0008043 impedes tumor growth *in vivo*. (a) The tumors after resection were photographed with a ruler. (b) Tumor weight was measured after resection. (c) Tumor volume was detected every 7 days from day 7 to 28 post-transfection. **P < 0.01.

Then, we explore the role of circ_0008043 *in vivo*. Knockdown of circ_0008043 significantly reduced the size and weight of xenograft tumors (Figure 5(a–c)).

### Circ_0008043 is a miR-326 sponge

**Figure 6.**
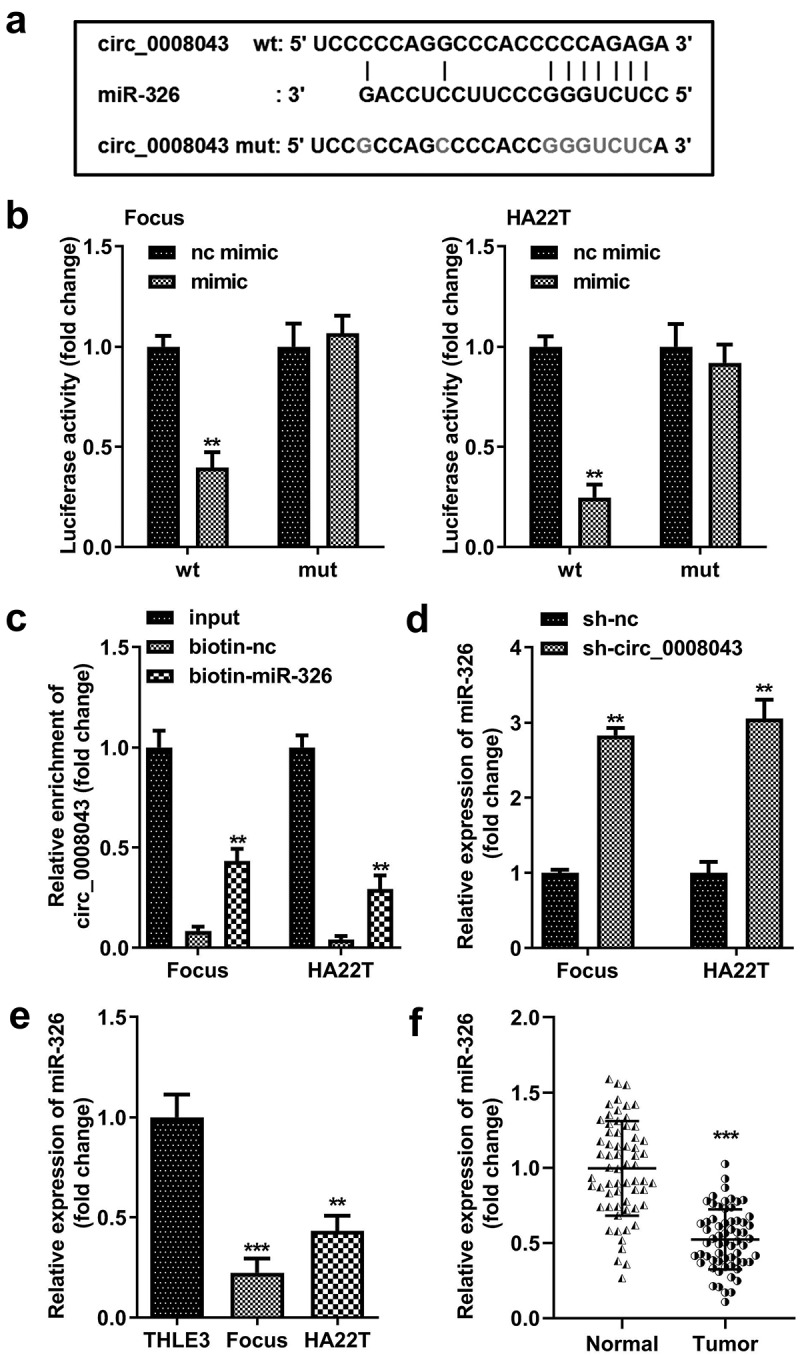
Circ_0008043 is a miR-326 sponge. (a) The sequences of wt circ_0008043 binding to miR-326 and the mut circ_0008043 sequences. The direct relationship between circ_0008043 and miR-326 was determined using (b) dual-luciferase reporter assay and (c) RNA pull-down assay. (d) MiR-326 expression was tested using qPCR following circ_0008043 knockdown. The miR-326 levels were tested using qPCR in (e) HCC/normal cells and (f) tumor/normal tissues. **P < 0.01. ***P < 0.001.

To understand the molecular mechanism, we predicted that miR-326 was a potential target of circ_0008043 (Figure 6(a)). The data from the luciferase reporter assay indicated that luciferase activity significantly decreased when co-transfected with the mimic and wt plasmids (Figure 6(b)).

Circ_0008043 was pulled down by biotin-labeled miR-326 (Figure 6(c)). Knockdown of circ_0008043 markedly upregulated miR-326 levels (Figure 6(d)). Moreover, miR-326 expression in HCC cells and tissues was significantly less than that in normal cells and tissues (Figure 6(e,f)).

### Circ_0008043 plays a tumor-promoting role in HCC by sponging miR-326

**Figure 7.**
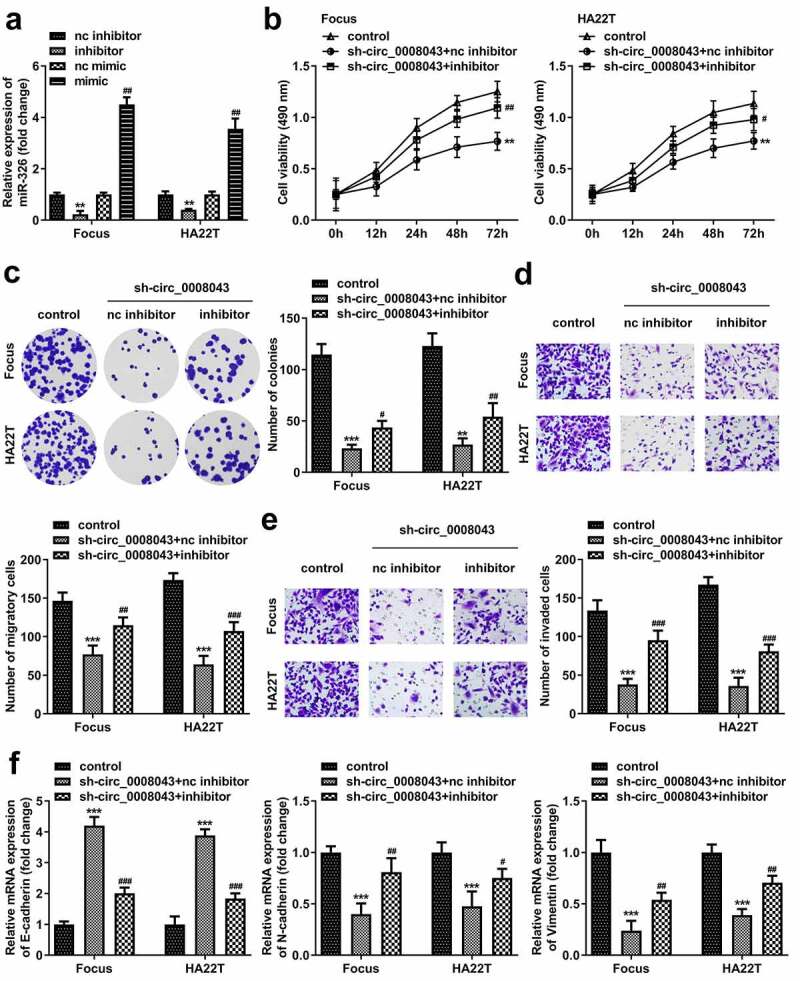
Circ_0008043 plays a tumor-promoting role in HCC by sponging miR-326. (a) MiR-326 expression was examined using qPCR following transfection with inhibitor or mimic. (b) MTT and (c) colony formation assays were conducted to evaluate cell proliferation. (d) Cell migration and (e) invasion were assessed using transwell assay. (f) qPCR was utilized to measure E-cadherin, N-cadherin, and vimentin expression. **P < 0.01. ***P < 0.001. #P < 0.05. ##P < 0.01. ###P < 0.001.

After transfection, the inhibitor markedly downregulated miR-326 expression, whereas the mimic markedly induced miR-326 upregulation (Figure 7(a)). miR-326 inhibition significantly abrogated the suppression of cell viability, colony formation, migration, and invasion induced by circ_0008043 knockdown (Figure 7(b–e)). Moreover, the increased E-cadherin level and decreased N-cadherin level, as well as vimentin expression induced by circ_0008043 loss, were significantly abolished by miR-326 inhibitor (Figure 7(f)).

### RAB21 is a miR-326 downstream target

**Figure 8.**
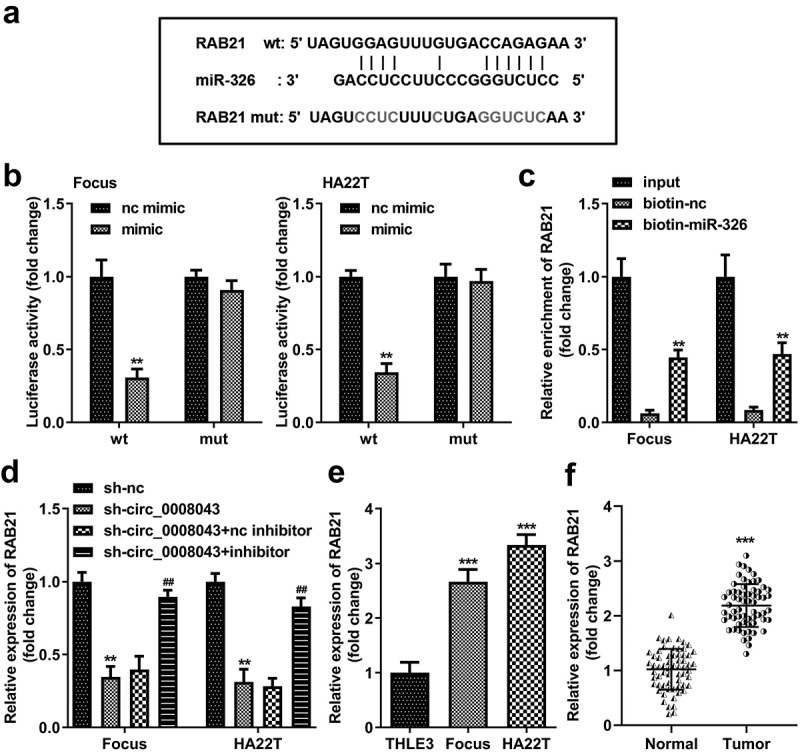
RAB21 is a miR-326 downstream target. (a) The wt RAB21 sequences binding to miR-326 and the mut RAB21 sequences are shown. (b) Dual-luciferase reporter assay and (c) RNA pull-down assay were performed to analyze the interaction between miR-326 and RAB21. (d) RAB21 expression was detected after downregulated circ_0008043 and miR-326 using qPCR. RAB21 was tested using qPCR in (e) HCC/normal cells and (f) tumor/normal tissues. **P < 0.01. ***P < 0.001. ##P < 0.01.

RAB21 was predicted to be an miR-326 target, according to the results of TargetScan database (Figure 8(a)). MiR-326 markedly reduced luciferase activity when co-transfected with wt plasmids (Figure 8(b)). Furthermore, biotin-miR-326 captured more RAB21 than biotin-nc (Figure 8(c)). Knockdown of circ_0008043 obviously decreased RAB21 expression, while inhibition of miR-326 rescued RAB21 expression (Figure 8(d)). RAB21 was significantly upregulated in HCC cells and tissues (Figure 8(e,f)).

### RAB21 rescues the miR-326 effect on HCC cells

**Figure 9.**
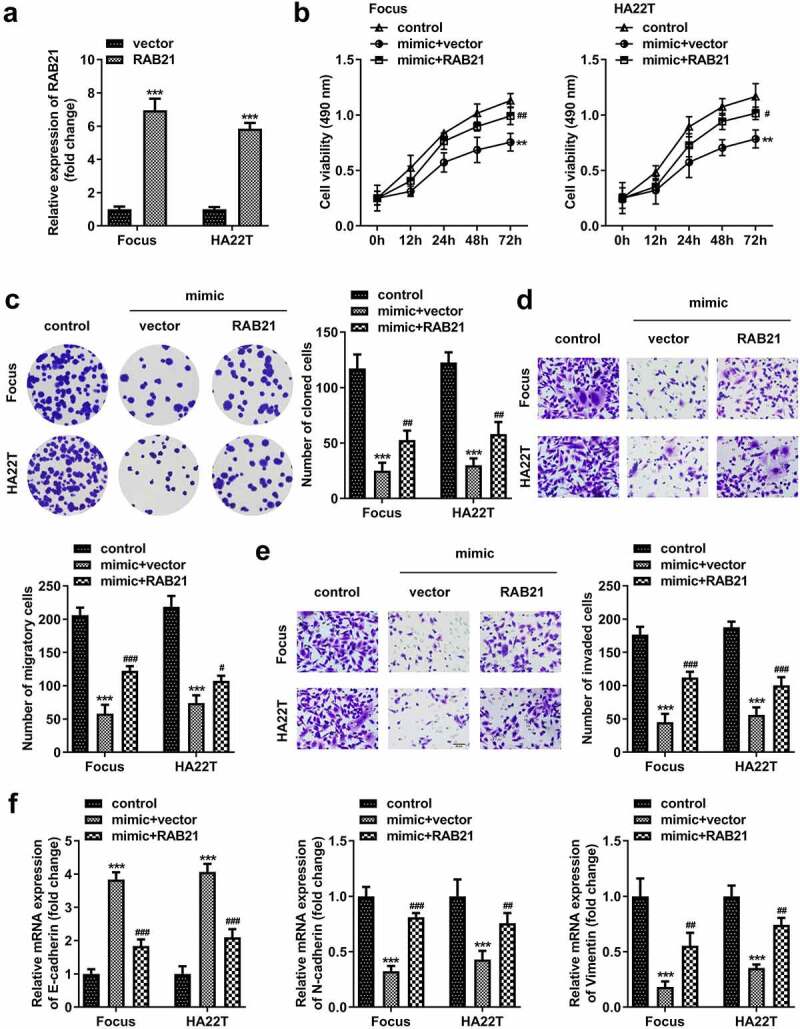
RAB21 rescues the miR-326 effect on HCC cells. (a) RAB21 expression was examined using qPCR post-transfection. (b) MTT and (c) colony formation assays were conducted to assess the proliferation. Transwell assay was used to assess (d) cell migration and (e) invasion. (f) qPCR was utilized to measure E-cadherin, N-cadherin, and vimentin levels. **P < 0.01. ***P < 0.001. #P < 0.05. ##P < 0.01. ###P < 0.001.

After transfection with RAB21-overexpressing vector, RAB21 levels were significantly elevated (Figure 9). RAB21 overexpression significantly abolished the suppression of proliferation, migration, and invasion induced by miR-326 (Figure 9(b–e)). MiR-326 significantly elevated E-cadherin and reduced N-cadherin and vimentin expression, while RAB21 abolished the miR-326 effect (Figure 9(f)).

## Discussion

In the present study, we found how circ_0008043 affected the progression of HCC. Moreover, circ_0008043 was confirmed to be an miR-326 sponge, which targeted RAB21. Circ_0008043 plays a role in HCC by regulating the miR-326/RAB21 axis.

An increasing number of circRNAs have been discovered in recent years, and several studies showed that they play a crucial role in the development of malignancy, including HCC. For example, circ_0001955 facilitates tumorigenesis, cell growth, and metastasis in HCC [29,30]. Hsa_circ_0091581 plays a role in ceRNA and promotes the malignant behavior of HCC by targeting miR-526b [31]. In addition, circ_0101432 is upregulated in HCC, and its depletion induces the suppression of proliferation and invasion [32]. However, the roles and mechanisms of abundant circRNAs remain unclear. In this study, we identified several dysregulated circRNAs according to microarray GSE155949 data. We confirmed that circ_0070467, circ_0025332, circ_0002141, and circ_0008043 were differentially expressed in HCC. Circ_0008043, with the most significant difference, was a stable circular transcript that was highly expressed in HCC cells and had a diagnostic value. Furthermore, interfering with circ_0008043 inhibited HCC progression. These findings demonstrate that circ_0008043 functions as an oncogene and has potential for HCC diagnosis and therapy. Circ_0008043 is a novel identified circRNA. It is the first study to explore the role of circ_0008043 in tumors.

To clarify the molecular mechanism, we found that circ_0008043 functions as a ceRNA to sponge miR-326. MiR-326 is a promising biomarker in tumor diagnosis, prognosis, and therapy. It is commonly downregulated in cancers and is associated with poor prognosis, rapid cell growth, promotion of metastasis, and unfavorable drug resistance [33]. MiR-326 contributes to reduce chemotherapy resistance of lung cancer cells [34]. Previous studies have revealed that decreased miR-326 expression suppresses proliferation and metastasis and inducing apoptosis of HCC cells, leading to the facilitation of tumor progression [35,36]. Similar to these findings, we also confirmed that miR-326 expression was reduced in HCC. Downregulation of miR-326 abrogated the effect of circ_0008043 inhibition on malignant behavior. The data demonstrated that miR-326 played a tumor-suppressing role in HCC, and circ_0008043 accelerated HCC progression by sponging miR-326. It is the first study that circ_0008043 directly sponges miR-326.

Furthermore, we found that RAB21 was a downstream candidate of miR-326. Interestingly, the role of RAB21 is contradictory in cancer. For example, RAB21 is enhanced in glioma tissues and cells, which knockdown inhibits the proliferation and induces apoptosis of glioma cells [24,37]. Inversely, RAB21 is downregulated in prostate cancer and suppresses tumor progression [26]. In addition, RAB21 could modulate multidrug resistance in cancer cells [38]. Thus far, the role of RAB21 in HCC has not been elucidated. Here, we found that RAB21 level was elevated in HCC, which overexpression rescued the miR-326 effect on cell proliferation, migration, invasion, and EMT. These data suggest that RAB21 may promote tumor progression, consistent with its role in glioma and prostate cancer [24,26]. Taken together, this study is the first to find that silencing of circ_0008043 decelerates the development of HCC via the miR-326/RAB21 axis.

However, there are still limitations of our study. Circ_0008043 could be diagnosis and therapy target. But it is a basic study, and a lot more research is needed before it can be used in the clinic. Our future work may focus the role of circ_0008043 and the circ_0008043/miR-326/RAB21 axis *in vivo*.

## Conclusion

Circ_0008043 acts as an oncogene in HCC. Negative regulation of HCC progression was affected by silencing of circ_0008043 by regulating the miR-326/RAB21 axis. These findings suggest that the circ_0008043/miR-326/RAB21 axis may have important clinical value in the treatment of HCC.

## Data Availability

The datasets used and analyzed during the current study are available from the corresponding author on reasonable request.

## Highlights

1. Circ_0008043 is highly expressed in liver cancer.
2. Interfering with circ_0008043 inhibits HCC cell migration and invasion.
3. Circ_0008043/miR-326/RAB21 axis promotes HCC progression.
